# Pulmonary nitric oxide in astronauts before and during long-term spaceflight

**DOI:** 10.3389/fphys.2024.1298863

**Published:** 2024-01-31

**Authors:** Lars L. Karlsson, Lars E. Gustafsson, Dag Linnarsson

**Affiliations:** Department of Physiology and Pharmacology, Karolinska Institutet, Stockholm, Sweden

**Keywords:** lung health, space flight, nitric oxide, planetary habitat atmosphere, gas density

## Abstract

**Introduction:** During exploratory space flights astronauts risk exposure to toxic planetary dust. Exhaled nitric oxide partial pressure (PENO) is a simple method to monitor lung health by detecting airway inflammation after dust inhalation. The turnover of NO in the lungs is dependent on several factors which will be altered during planetary exploration such as gravity (G) and gas density. To investigate the impacts of these factors on normal PENO, we took measurements before and during a stay at the International Space Station, at both normal and reduced atmospheric pressures. We expected stable PENO levels during the preflight and inflight periods, with lower values inflight. With reduced pressure we expected no net changes of PENO.

**Material and methods:** Ten astronauts were studied during the pre-flight (1 G) and inflight (µG) periods at normal pressure [1.0 ata (atmospheres absolute)], with six of them also monitored at reduced (0.7 ata) pressure and gas density. The average observation period was from 191 days before launch until 105 days after launch. PENO was measured together with estimates of alveolar NO and the airway contribution to the exhaled NO flux.

**Results:** The levels of PENO at 50 mL/s (PENO50) were not stable during the preflight and inflight periods respectively but decreased with time (*p* = 0.0284) at a rate of 0.55 (0.24) [mean (SD)] mPa per 180 days throughout the observation period, so that there was a significant difference (*p* < 0.01, N = 10) between gravity conditions. Thus, PENO50 averaged 2.28 (0.70) mPa at 1 G and 1.65 (0.51) mPa during µG (−27%). Reduced atmospheric pressure had no net impact on PENO50 but increased the airway contribution to exhaled NO.

**Discussion:** The time courses of PENO50 suggest an initial airway inflammation, which gradually subsided. Our previous hypothesis of an increased uptake of NO to the blood by means of an expanded gas-blood interface in µG leading to decreased PENO50 is neither supported nor contradicted by the present findings. Baseline PENO50 values for lung health monitoring in astronauts should be obtained not only on ground but also during the relevant gravity conditions and before the possibility of inhaling toxic planetary dust.

## Introduction

Inhalation of toxic dust is a potential risk factor during future manned space exploration and therefore it is essential that lung health is monitored (Linnarsson et al., 2012; Pohlen et al., 2022). Future space vehicles and habitats for human planetary exploration are expected to have an atmosphere with reduced atmospheric pressure, and the associated reduced gas density will impact certain tests that need to be performed to monitor the lung health of the crewmembers. In addition to classical spirometry, exhaled nitric oxide (NO) can be used to monitor airway inflammation after, for example, toxic dust inhalation on the Moon or Mars (Gustafsson et al., 1991; Alving et al., 1993; Pietropaoli et al., 2004; Linnarsson et al., 2012). Airway NO is formed in both alveolar and conducting airways, and its concentration in exhaled air depends not only on its rate of formation, but also on how much of the NO is transported by back-diffusion to the alveoli and then taken up by the blood (Kerckx et al., 2008). In a previous study, we have found that ambient pressure and gas density alter certain basic parameters of the pulmonary NO turnover, likely because of density-induced changes in the balance between diffusive and convective gas transport in the lung periphery (Hemmingsson et al., 2012).

During planetary exploration gravity will be different from that on earth, with the Moon having 16% of Earth’s gravity, Mars 38%, while weightlessness will be experienced during the long periods required for transfer between celestial bodies. We have previously found that baseline (non-inflammatory) exhaled NO is markedly reduced in microgravity (Karlsson et al., 2009a). Thus, the exhaled NO fractional concentration was reduced by 46% during the first week on the International Space Station (ISS) as compared with pre-flight controls on earth. We further found increased exhaled NO in humans exposed to hypergravity in a human centrifuge (Karlsson et al., 2009b; Kerckx et al., 2010). Based on these findings we concluded that an increase of the area of the blood/gas interface in weightlessness (Prisk et al., 1993; Verbanck et al., 1997; Prisk, 2014; Karlsson et al., 2023) and a corresponding increase of NO uptake to the blood resulted in less exhaled NO. The reverse process (Rohdin and Linnarsson, 2003; Karlsson et al., 2009a) was proposed to take place in hypergravity, leading to an increase in exhaled NO.

Together, the above studies investigating the effects of atmospheric pressure and gravity on exhaled NO show that baseline (pre-inflammatory) values, when obtained in space and during transport to a planetary habitat, are likely to change compared to the corresponding values obtained preflight on ground in normal gravity and atmospheric pressure. If so, the ground control values will no longer be useful as reference values for lung health monitoring during exploration space flights but must be reassessed inflight. The present study was undertaken to quantify the impact of the space-flight environment as such on a standard test for exhaled NO. Astronauts were studied before and during a stay at the International Space Station (ISS) in normal and reduced atmospheric pressure. Based on earlier studies (Kerckx et al., 2008; Karlsson et al., 2009b; Hemmingsson et al., 2012; Karlsson et al., 2023) we expected lower values of exhaled NO in microgravity than on ground, and alterations in the balance between alveolar and conductive-airway contributions to exhaled NO with changes in atmospheric pressure. Moreover, for this group of subjects we expected stable values within the normal range during the preflight period, and lower but equally stable values inflight.

## Methods

### Ethics

The research was approved by the Regional Research Ethics Board of Stockholm nr 2009/1341-31/3. Corresponding approvals were obtained from the internal review boards of the European Space Agency (ESA), the US National Aeronautics and Space Administration (NASA) and the Japanese Space Agency (JAXA). The subjects received both oral and written information about the procedures and their scientific background, and written consents were obtained, and the subjects were aware of their right to withdraw from the experiment without prejudice at any time.

### Subjects

A group of 10 astronauts were studied, seven males and three females. Their age, height and body mass ranged from 37 to 51 years, 1.65–1.85 m and 63–89 kg, respectively. They all declared themselves healthy and without a history of inflammatory airway disease. The volunteers underwent a screening test with determination of exhaled nitric oxide at a flow of 50 mL*s^−1^ according to the standards of American Thoracic Society and European Respiratory Society (American Thoracic Society and European Respiratory Society, 2005), and a value no higher than 35 parts per billion (ppb) was required for inclusion.

### Experimental conditions

Experiments in normal gravity [1 G, baseline data collection (BDC)] took place at Johnson Space Center, Houston, TX, United States, except on two occasions at the European Astronaut Centre, Cologne, Germany and one occasion at Skrydsrup Air Force Base, Denmark. Procedures were performed at normal atmospheric pressure (1.0 atm absolute, 1.0 ata) and at 0.7 ata in hypobaric chambers, the latter pressure being that found at an altitude of 3,000 m (9,900 ft). The experiments in weightlessness (microgravity, µG) were performed on the ISS at normal pressure, and at 0.7 ata in the United States Air Lock, which is normally used for preparations before extravehicular activity (EVA, spacewalks) (Table 1). Subjects breathed air during the 1.0 ata experiments and a gas mixture with 27.5 per cent oxygen in nitrogen at 0.7 ata. This percentage was a compromise between the desire to avoid hypoxia and at the same time to keep the oxygen fractional concentration below the threshold specified for certain equipment in the United States Air Lock. The resulting inspired oxygen partial pressure at 0.7 ata corresponds to an altitude of 700 m (2,300 ft) breathing air.

**TABLE 1:** Environmental conditions.

Condition	Pressure, hPa		Cabin PO2, kPa		Temperature, ^o^C		Rel. Humidity, %	
	Mean	SD	Mean	SD	Mean	SD	Mean	SD
1 G, 1.0 ata	1013	7	21.2	0.1	22.4	1.6	68	10
1 G, 0.7 ata	707	4	19.4	0.1	23.2	1.2	63	6
µG, 1.0 ata	1003	10	21.0	0.2	24.2	1.0	40	2
µG, 0.7 ata	700	8	19.3	0.2	23.9	1.4	43	4

Rel, relative.

Each astronaut participated in two to four 1 G sessions during the pre-flight period, while nine of the 10 subjects took part in two µG sessions on the ISS, whereas one subject only had one µG session. The first and last 1 G sessions occurred during days −191 (59) [Mean (SD)] and −82 (23) before launch. The corresponding days for the µG sessions were +36 (17) and +105 (33) after the launch. Out of the two µG sessions one, was performed at 1.0 ata only and one at both atmospheric pressures.

### Equipment

Exhaled NO was measured with the same type of analyser (Niox Mino, Aerocrine AB, Solna, Sweden/Circassia PLC, Oxford, United Kingdom) as in our earlier experiments on the ISS (Karlsson et al., 2009a). The analyser was further equipped with a set of resistors and a manifold that permitted measurements at three different exhaled flows, approximately 30, 50, and 150 mL*s^−1^ when the subject applied an airway pressure of 15 cmH_2_O, as guided by visual feed-back on a screen. The elevated airway pressure acted to close the soft palate, thereby preventing contamination with NO from the nasal cavity. Using the same set of resistors at 0.7 ata the corresponding flows were 14%–17% higher than at 1.0 ata due to the lower gas density. The analyser and the flow control system had been adapted for use on the ISS by the Danish Aerospace Company (DAC), Odense, Denmark. Four sets of equipment were manufactured, one flight unit and three units for testing and BDC on earth. The pressure/flow characteristics of the flow control system of the four units were reconfirmed on ground after the experiments. Data for airway pressure, exhaled NO and manifold setting were obtained continuously and stored in dedicated data acquisition systems on the ISS and the ground (Portable Pulmonary Function System, DAC, Odense, Denmark). Data were also stored for atmospheric pressure, room/cabin temperature, relative humidity, and serial number of the individually calibrated sensor element of the NO analyser. The progress of the experiments was monitored by the investigators on site during BDC and online in a ground control facility at DAC during experiments on the ISS.

### Procedures

Nitrite and nitrate-containing food may influence exhaled NO and was therefore not allowed to be consumed 12 h before a session. Examples of such food are sausages, ham, and green vegetables such as beets, spinach, and lettuce. The subjects rinsed their mouth with water before each experiment to minimize contamination of the exhalate with traces of NO from mouth bacteria. No EVA or EVA training was permitted 24 h before an experiment, as high oxygen partial pressures may potentially influence airway NO. A complete list of constraints can be found in Supplementary Material.

The standard NO measurement maneuver was as follows: an initial deep exhalation to the room/cabin air, followed by a full inhalation of NO-free air through a filter in the analyser, and finally a 16 s long slow exhalation from which an NO sample was obtained. At 1.0 ata subjects performed three maneuvers at each flow in random order and at 2–3 min intervals, whereas at 0.7 ata there were four repetitions at each flow.

### Data analysis

**FIGURE 1:**
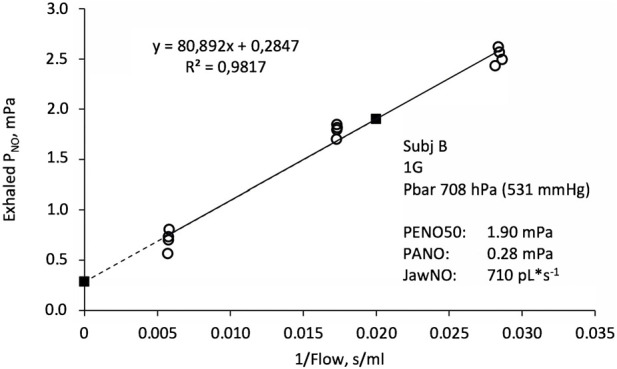
Exhaled NO partial pressure (PNO) as a function of the inverse of exhaled flow. Open circles indicate original data from four exhalations in each of the three flow ranges, together with a fitted linear function. Filled squares are estimates of the alveolar PNO (PANO, left) and PNO at an exhaled flow of 50 mL*s−1 (PENO50, right). The slope of the fitted function is proportional to the conductive airway contribution to the exhaled NO flux (JawNO).

All readings of exhaled NO were converted to partial pressure using calibration factors that had been determined for each individual sensor at both 1.0 and 0.7 ata. For NO, as for other metabolic gases such as CO_2_, fractions are misleading when comparing results obtained at different atmospheric pressures (Hemmingsson et al., 2012). Partial pressures of exhaled NO (PENO) were then plotted as a function of the inverse of the exhaled flow (body temperature and pressure, saturated with water vapour, BTPS) the latter obtained from recordings of airway pressure combined with a pressure/flow calibration factor determined for each individual resistor and ambient pressure. Figure 1 shows a typical data set. This graphical representation is based on a simple two-compartment model of the NO turnover in the lung (Pietropaoli et al., 1999; Hemmingsson et al., 2012), in which gas from an alveolar compartment takes up additional NO from the conductive-airway epithelium during exhalation. From the fitted linear relationship between PENO and 1/flow, three parameters were extracted: a) PENO at 50 mL*s^−1^ (PENO50), b) PENO at the intercept of the *Y*-axis as an estimate of the alveolar NO partial pressure (PANO), and c) the slope of the fitted function, which after conversion by a factor (10000*273)/(1013*307) is an estimate of the NO contribution from the conducting airways (JawNO) in pL*s^-1^ standard temperature and pressure, dry (STPD).

Statistics: Temporal trends of the three parameters at normal pressure were analyzed using a linear mixed effects model. Differences between pressures were analysed with ANOVA in a subset of the subjects (see below). The level for statistical significance was set to *p* < 0.05.

## Results

Due to a combination of technical malfunctions, time constraints onboard the ISS and limitations of uploading of supplies, complete data sets were only obtained for six of the ten subjects. For the first two subjects to be studied onboard the ISS, a previously unobserved malfunction of the NO sensors after decompression precluded the 0.7 ata measurements. This problem was solved for the next six subjects by testing the speed of adaptation to decompression in a large number of sensors and selecting the approximately one out of 10 that adapted quickly for uploading to the ISS. For the last two subjects the timing of the experiments was changed so that timely uploading of the necessary sensing elements was not possible. At 1.0 ata, however, complete data sets were obtained for all subjects. A study of lung diffusing capacity for NO (DL_NO_) was performed in parallel to the present experiments (Karlsson et al., 2023).

### Quality of data

The manufacturer of the NO analyser used in the present study specifies a precision of <3 ppb (<0.3 mPa at sea level) below 30 ppb for single point PENO50 determinations. Each PENO50 value is based on 9–12 PENO determinations (see Figure 1), so a conservative estimate of the precision of the present PENO50 data will be <0.1 mPa. Generally, the fit of experimental data into the linear function shown in Figure 1 was good with a median *R* ^2^ value of 0.975.

### Exhaled NO and related parameters at normal pressure

**FIGURE 2:**
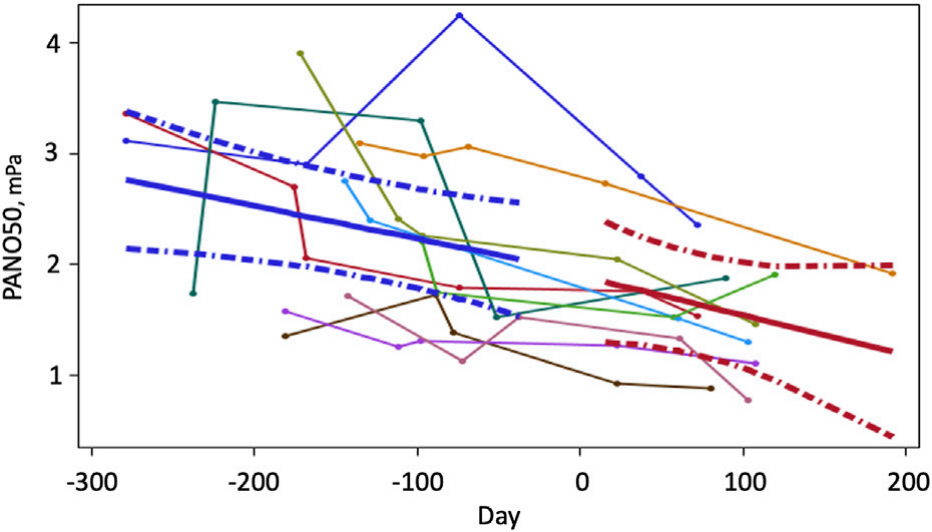
PENO50 as a function of time (*N* = 10) before and during a prolonged stay at the international space station. Time zero is the launch date. Data were obtained between two to four occasions before launch and twice on the ISS. Thin lines represent time series of individual values. Solid heavy lines are mean trends and broken lines show 95% confidence limits for the fitted lines.

Two trends were obvious in the data obtained for PENO50 during the preflight and inflight periods (Figure 2): First there was a great variability of PENO50 with time and between subjects during the preflight period. The other was that despite this variability, there was a significant decrease of PENO50 with time (*p* = 0.0284) at an average rate of 0.55 (0.24) mPa per 180 days throughout the observation period. There was also a significant difference (*p* < 0.01, *N* = 10) between gravity conditions with a mean PENO50 of 2.28 (0.70) mPa at 1 G and 1.65 (0.51) during µG (−27%).

Seven of the 10 subjects showed reasonably stable decreases with time. Even though all subjects were screened to have PENO50 values no larger than 3.5 mPa (35 ppb at sea level pressure) before the study, higher values were observed on two occasions during the pre-flight period. The three subjects with the largest variability were all male (*R* ^2^ < 0.2), whereas the three female subjects showed reasonably linear trends (*R* ^2^ > 0.7).

To deal with the variability, we applied the linear mixed effect model multiple (10000) times using a bootstrap with replacement approach. The 95% confidence limits for the slope of PENO50 (−0.817 and −0.378 mPa/180 days, *p* < 0.001) did not include zero, which demonstrates the robustness of our findings despite the variability.

For the estimate of alveolar NO (PANO) there was a similar trend as for PENO50. Thus, PANO showed a significant decrease with time (*p* = 0.0043) at a mean rate of 0.28 (0.09) mPa per 180 days. There was also a significant difference (*p* < 0.01, *N* = 10) between gravity conditions so that mean PANO was 0.61 (0.25) mPa at 1 G and 0.33 (0.12) during µG. Corresponding values for JawNO were 765 (272) and 577 (238) with no significant trend in terms of time.

### Effects of ambient pressure

**FIGURE 3:**
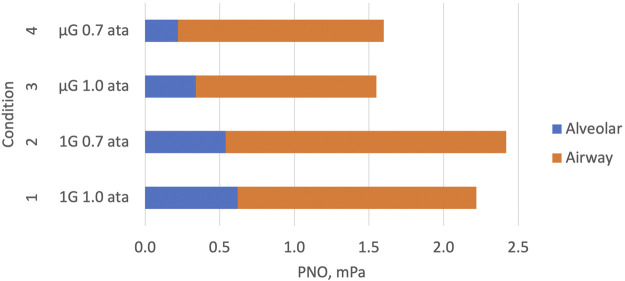
Graphical representation of the key elements of Table 2. Components of exhaled NO (PENO50) are shown during four combinations of normal gravity (1G), microgravity (µG), normal atmospheric pressure (1.0 ata) and 30% reduced atmospheric pressure (0.7 ata). Data for the alveolar and conductive airway components of PENO50 have been estimated with the two-compartment model described in Figure 2. The conductive-airway component is proportional to the slope of the linear relationship shown in Figure 2. Attenuated NO formation and/or enhanced alveolar uptake to the blood leads to reduced alveolar and exhaled NO in µG. At 0.7 ata the conductive-airway component is enhanced compared to 1.0 ata, probably due to attenuated back-transport from the conductive airway segment to the alveolar segment.

Data pertaining to the effects of ambient pressure could be studied in a subset of the subjects and are shown in Table 2; Figure 3. Inflight, experiments at 0.7 ata were only performed at one point in time. All other data are mean values for all experiments performed at normal G and µG respectively.

**TABLE 2:** Group mean values (*N* = 6) and standard deviations of exhaled NO partial pressure at a flow of 50 mL*s−1 (PENO50), the conductive-airway contribution to the exhaled NO flux (JawNO), and the alveolar NO partial pressure (PANO) during four combinations of gravity (1G, normal gravity; µG, weightlessness) and pressure (atmospheres absolute, ata). *p* values in the three right-hand columns refer to effects of gravity (G), pressure (P), and gravity-pressure interaction, respectively, where bold numbers indicate a significant effect.

	Gravity	Pressure, ata	Mean	SD	G effect	P effect	G *P
PENO50 mPa	1G	1.0	2.22	0.72	**0.0095**	0.1204	0.4060
		0.7	2.42	0.65			
	µG	1.0	1.55	0.48			
		0.7	1.60	0.57			
JawNO pL*s^-1^	1G	1.0	703	264	0.0556	**0.0224**	0.4450
		0.7	825	251			
	µG	1.0	531	211			
		0.7	607	217			
PANO mPa	1G	1.0	0.62	0.26	**0.0048**	0.0546	0.7130
		0.7	0.54	0.22			
	µG	1.0	0.34	0.13			
		0.7	0.22	0.14			

At normal pressure, the mean values of the three parameters for this subset of the subjects were almost identical to those of the larger group. There was a significant effect of pressure on JawNO with a 16% increase at reduced pressure, and at the same time a trend for PANO to be lower than at normal pressure. There was also a significant G effect on PANO with 56% lower values in µG compared to normal G.

## Discussion

### Lower exhaled NO in microgravity

The main finding of the present study is that a standard test for exhaled NO (PENO50) showed gradually decreasing values over an extended pre-flight period, which then continued to fall at a similar rate during the stay on the ISS. This contrasts with our previous observations (Karlsson et al., 2009b) where no trends were observed during the 10–30 days long pre-flight period, and a drop of PENO50 to half the pre-flight level was observed the first week in µG, with no further change during the 6 months stay on the ISS. The timing of the testing was different between the two studies, and the relatively short pre-flight observation period in our previous study likely precluded detection of any time trends. Another important difference between the two studies was that the first measurement of PENO50 on the ISS in the present study took place after an average period of 37 days. Therefore, even if there would be a distinct reduction of PENO50 related to the transition into µG, it could not have been detected. Our data from a study of DL_NO_ in the same subjects and conditions (Karlsson et al., 2023) showed that this index of the efficiency of the blood-gas interface was increased in microgravity, but to a much lower degree than similar measurements made by others during the first week or weeks of microgravity (Prisk et al., 1993; Verbanck et al., 1997). Again, if an expanded gas blood interface enhances back diffusion and blood uptake of NO, it cannot be excluded that PENO50 could have been even lower during the initial period than the presently observed values, which were obtained after a longer period of microgravity.

Another striking difference between our previous study (Karlsson et al., 2009a) and the present study is the level of PENO50 during the pre-flight period, which had a mean of 1.2 mPa (12 ppb at 1.0 ata) in the previous study and almost double the value (2.28 mPa) in the present study. A predicted value weighted for age, gender, and ethnic background (Jacinto et al., 2018) for the present group of subjects would be 1.33 mPa (13,3 ppb at 1.0 ata). Six of the 10 subjects showed values above the upper 95 percentile (Jacinto et al., 2018) at one or more occasions during the pre-flight period, i.e., values above 3.3–3.5 mPa in the males and 2.5 mPa in the females. It should be added that the present maximum permitted value for inclusion (3.5 mPa) was established in 2009, well before Jacinto et al. (2018) and other research groups had presented more accurate predictions and upper limits.

The above findings were unexpected and raise questions about whether an element of airway inflammation was present initially and then gradually subsided. The similarity between the trends for PENO50 and PANO support the notion that both conductive and alveolar airways are involved. Unfortunately, no allowance for detailed studies of subject health were included in the approved experimental plan, other than self-reporting of acute symptoms of respiratory tract infections and assurances from the subjects that they had no history of inflammatory airway disease. Thus, the elevated PENO50 values in many of the subjects during the early pre-flight period and the significant trend of a decrease over the following year including the inflight period deserve further study. The higher-than-normal mean value in the group could have been caused by local environmental factors, such as the periodically poor air quality in the greater Houston area (Banta et al., 2005; Luke et al., 2010). It is further not unreasonable to assume that the time trend would be related to the regime during the pre-flight period including lifestyle changes imposed by the training program. It may be speculated that the EVA training with hours of breathing of oxygen-enriched gas mixtures could have had an impact even if there was a specified 24 h time limit between EVA training or actual EVA and the present experiments. On the other hand, an earlier study from our laboratory of simulated and actual EVA (Karlsson et al., 2009a) showed no acute effects on PENO50. The continued trend of decreasing PENO50 during the stay on the ISS could represent a further recovery from environmental factors on ground.

### Effects of ambient pressure and gas density

Hemmingsson et al. (2012) studied the effects of ambient pressure and gas density on PENO50, PANO and JawNO over a much larger range (0.5–4.0 ata, gas density 0.6–4.8 g/L) than in the present study (0.7–1.0 ata, gas density 0.8–1.2 g/L). Their experiments were conducted at normal gravity. Based on the concept of competition between the amount of exhaled NO on one hand and that taken up by the blood after back diffusion on the other (Kerckx et al., 2008) they expected that facilitated back diffusion with reduced gas density would lead to a reduction of JawNO and an increase of PANO. Instead, Hemmingsson et al. (2012) found the opposite and proposed that the back transport of NO from its site of formation in conducting airways to the alveoli is a function of both convective and diffusive processes, in which the reduced gas density seems to impair convective penetration of gas to the periphery more than it facilitates diffusion. As a net result, exhaled NO as reflected by PENO50 was strikingly similar over this wide range of gas densities, whereas the conductive airway contribution to exhaled NO (JawNO) was increased and alveolar NO decreased with reduced gas density.

The present findings at reduced pressure (Table 2; Figure 3) were in line with those of Hemmingsson et al. (2012) with no significant pressure effect on PENO50, a significant pressure effect on JawNO with a 16% increase, and a concomitant trend for a decreased PANO, all compared to normal pressure. A tentative explanation for the latter finding may be found in the DL_NO_ data obtained at the same time (Karlsson et al., 2023): a model for the resistance of NO transport in the small airways of the lung periphery suggested that this resistance was markedly increased in µG due to tissue edema, resulting in larger NO partial pressure differences along these airways than in normal G. A similar phenomenon in the airway segment between the site of NO formation and the alveolo-capillary membrane could have contributed to the trend for a lower PANO with reduced pressure. In summary, therefore, our data obtained at 0.7 ata suggest that the exhaled NO then originates less from the alveoli and more from the conductive airways compared to exhaled NO at normal pressure.

### Limitations

#### Experimental conditions

Our initial goal was to perform studies in a moderately hypoxic atmosphere at 0.5 ata, which were the conditions foreseen in future planetary habitats (NASA, 2010). However, concerns that hypoxia might worsen the Spaceflight Associated Neuro-ocular Syndrome (Mader et al., 2011) motivated a change to the present atmospheric conditions. For further details, see Karlsson et al., 2023. Further experiments at the appropriate pressure and oxygen concentration are thus required for determination of how lung function tests such as exhaled NO will be influenced by the combination of altered gravity and atmosphere in future planetary habitats.

There was a small difference in cabin oxygen partial pressure between the experiments at 1.0 and 0.7 ata (Table 1). This difference is not likely to have influenced the PENO50 results given that in a meta study of seven trials MacInnis et al. (2015) showed that normobaric hypoxia had no influence on exhaled NO.

Table 2 also shows a systematic difference between the relative humidities (RH) during the ground experiments and those performed on the ISS. The manufacturer of the NO analyser had specified a minimum humidity of 30% and humidity was always above that. The above difference in RH was anticipated, so as an extra precaution all sessions started with ten exhalations into the device to make sure that its inner surfaces were humidified to a consistent degree throughout the study.

#### Number of subjects in different parts of the study

The unfortunate combination of technical problems and operational constraints dictated that complete data for both atmospheric pressures could only be obtained from six of the 10 subjects. However, at 1.0 ata, group mean data for exhaled NO and related parameters were almost identical between the *N* = 6 and *N* = 10 groups and this makes it likely that data from the subset can be considered representative also for the larger group. Moreover, *N* = 6 is common in inflight studies on astronauts whereas *N* = 10 is an uncommonly large group in such a setting.

## Conclusion

In summary, our previous hypothesis of an increased uptake of NO to the blood by means of an expanded gas-blood interface in µG leading to decreased PENO50 (Karlsson et al., 2009b) is neither supported nor contradicted by our present findings. Also, our initial hypothesis for the present work with stable but different PENO50 levels before and during µG was not confirmed. Instead, we found a large variability of exhaled NO in some of the astronauts during the preflight period, and a general trend of gradually decreasing values during the whole observation period including the inflight period. The observed time trend with gradual reductions of both PENO50 and PANO suggests an element of airway inflammation, which gradually subsides during the observation period including the stay on the ISS. However, even if the rate of decrease seems to be the same before and after the transition into µG the mechanisms may have differed. A 30% reduction of ambient pressure and gas density showed no net effect on the exhaled NO partial pressure, but was accompanied with a higher conductive airway contribution to the exhaled NO. A practical conclusion for lung health monitoring during future exploration space flights is that baseline values for PENO should be obtained not only on ground but also in the relevant gravity environment and before there is a risk for inhalation of toxic planetary dust. Finally, the general trend with higher PENO50 values initially during the preflight period followed by a gradual fall deserves further studies. A more clinically oriented approach would in our view be required to identify the factors behind the present PENO50 findings.

## Data Availability

The datasets presented in this article are not readily available because while the European Space agency (ESA) seeks to maintain the scientific value of the raw data and to enable research, ESA must balance this with the protection of the astronaut’s right to privacy applicable under the ESA Protection of Personal Data Framework and as applicable, the GDPR, which both override national law. Due to the limited number of astronauts and the fact that their specific activities (date, time, place, gender) make them easily identifiable in person, raw data cannot be distributed as it would involve unreasonable effort to de-identify and anonymise such data. Requests to access the datasets should be directed to lars.karlsson@ki.se.
