# Supplementary material for: Pulmonary nitric oxide in astronauts before and during long-term spaceflight
Source: Front Physiol. 2024 Jan 31;15:1298863. doi: 10.3389/fphys.2024.1298863 (PMC10864557; doi:10.3389/fphys.2024.1298863)
Supplement: Supplementary file 1 [file Presentation1.pdf]

Supplementary material

CONSTRAINTS REGARDING PARTICIPATION AND SCHEDULING IN THIS STUDY (NAMED "AIRWAY MONITORING")

1 Scheduling and Session Constraints

1.1 Extravehicular Activity (EVA)

No sessions on the day of an EVA.

1.2 Exercises

No exercises during two hours prior to Airway Monitoring testing. No strenuous exercises (75% VO2 max or higher – corresponding to heart rate 150 bpm or higher) 24 hours prior to test.

1.3 Scuba diving and T-38 flying

No scuba diving or T-38 flying 24 hours prior to Airway monitoring BDC.

1.4 Sleep Shifting

No sleep shifting of more than two hours during the 24 hours prior to Airway Monitoring testing (pre- and inflight).

1.5 Airplane travel

No flying on the day of Airway Monitoring BDC prior to testing (BDC only).

1.6 Crew health

If a crewmember has a cold, the Airway Monitoring session should be delayed. The astronaut should be free of any (cold) symptoms for at least three days prior to measurements.

1.7 Diet

Nitrite and nitrate- containing food is not allowed 12 hours before a session. Examples of such food are sausages, ham, liver pate, and green vegetables such as beets, spinach and lettuce (fresh meats, fresh eggs and cheese are OK).

2 Constraints regarding other experiments

Potential constraints against experiments that require pharmacological interventions and/or a special diet to be consumed.
